# The Effectiveness of Smoking Prevention Module Towards Knowledge and Smoking Refusal Skills among Adolescents in Kota Bharu, Kelantan, Malaysia

**DOI:** 10.31557/APJCP.2019.20.11.3353

**Published:** 2019

**Authors:** Norlina Anuar, Nur Suhaila Idris, Faridah Mohd Zin, Razlina Abdul Rahman, Imran Ahmad, Mohd Ismail Ibrahim

**Affiliations:** 1 *Department of Family Medicine, *; 2 *Department of Community Medicine, School of Medical Sciences, Universiti Sains Malaysia, Kelantan, Malaysia. *

**Keywords:** Smoking prevention, knowledge, adolescent, refusal to participate

## Abstract

**Objective:**

To study the effectiveness of the smoking prevention module towards knowledge on smoking and its harmful effects and smoking refusal skills among secondary school students in Kelantan, Malaysia.

**Methods:**

A quasi experimental interventional study involving 166 non-smokers adolescents, aged 13 to 14 years old were carried out in two schools located in two different suburbs. Both schools had equal number of participants. One school was given the smoking prevention module for intervention while the control school only received the module after the study had been completed. The knowledge on smoking and its harmful effects and smoking refusal skill score were assessed using a set of validated Malay questionnaires at baseline, two weeks and eight weeks after the intervention. Repeated measure ANCOVA was used to analyse the mean score difference of both groups at baseline and after intervention.

**Result:**

Baseline analysis shows no significant difference in knowledge score between the study groups (p = 0.713) while post intervention, it shows significant inclination of knowledge score in intervention group and the difference was significant after controlling the gender [F(df) = 15.96(1.5), p <0.001]. The mean baseline for refusal skills score in the control and intervention groups were 30.89(6.164) and 28.02(6.241) respectively (p= 0.003). Post intervention, there is a significant difference in the crude mean and the estimated marginal means for smoking refusal skills score between the two groups after controlling for sex [F(df) = 5.66(1.8), p = 0.005].

**Conclusion:**

This smoking prevention module increased the level of knowledge on smoking and its harmful effects and smoking refusal skill among the secondary school students. Thus, it is advocated to be used as one of the standard modules to improve the current method of teaching in delivering knowledge related to harmful effects of smoking and smoking refusal skill to the adolescents in Malaysia.

## Introduction

Smoking or tobacco use is one of the preventable causes of mortality in Malaysia. It is known that smoking is a risk factor for a wide range of chronic diseases such as cardiovascular disease and peripheral vascular disease (Thyrian et al., 2009). Additionally, the non-smokers are also getting detrimental effects from smokers. Breathing the environmental tobacco smoke may increase risks of getting lung cancer, chronic lung disease, respiratory infections and cardiovascular disease. Unfortunately, most of the adolescents are not aware of these harmful consequences of the activity, making them susceptible prey to initiation of smoking (Freedman et al., 2011).

Ministry of Health, Malaysia is directed towards reducing the smoking prevalence to half by year 2020 to reduce its disease related effects (Hock et al., 2013). Multiple initiatives had been taken to ensure that Malaysia is able to achieve the aforementioned target. These initiatives are crucial to avert smoking initiation at an early age as 80% of adult smokers admitted that they had started smoking since adolescence, while those who had not smoked during schooling years are less likely to commence smoking during adulthood (Hock et al., 2013). Preventive strategies are important since studies have shown that smoking cessation is difficult once the habit had started and will gradually become a long-term addiction (Gervais et al., 2006; Hock et al., 2013). 

Despite the multitude of campaigns and programmes, the results have remained unclear and the success rates are not impressive (Park and Drake, 2015). Despite various efforts to reduce the rate of adolescent smoking, it appears that the number of teens who smoke is escalating (Norbanee et al., 2006). There are multiple factors influencing the decision of the adolescent to be involved in smoking activities. Besides the efficacy of the prevention programmes, their self-interest also acts as a determinant whether these adolescents will engage in any smoking activities or otherwise. It is quite difficult for the adolescents, especially those with low self-esteem, to steer clear from smoking when it is recognised as socially enviable behaviour (Simons-Morton et al., 1999). 

Teaching refusal skills to adolescents will increase their ability to refuse peers’ invitation to be involved in smoking activity. It is rather challenging for adolescents to refuse cigarettes as smoking fulfills a social role which outbalances the health risks they are exposed to when they smoke (Charlton et al., 1999). Poor refusal skills are commonly associated with the increase in the likelihood of adolescents to partake in smoking activity (Gibbon et al., 2014). Adults and adolescents normally have different perceptions with regard to life issues, including how to say no to smoking invitation. There are a few methods of refusing which were deemed effective and could be applied by the adolescents such as making a firm statement against the behavior (“*I do not smoke!*”), making a simple statement (“*no thanks*”), giving an excuse (“*it’s illegal to smoke*”), changing the issue of the discussion or invitation (“*did you watch the last night’s show?*”) or non-verbal response, such as walking away from the group without saying anything (Nichols et al., 2010). 

It is imperative that any module is tested to ensure that the effort is not futile and the outcome is as expected. In this study we adapted an existing module used in national school and improved it based on local and international experience, keeping the modification suited to the local adolescents. 

## Materials and Methods

*Setting and samples*

The study was conducted in two secondary schools from two different suburbs in Kota Bharu district between August 2016 and October 2016. The schools which were located about 20 kilometres apart were purposely selected to reduce risk of contamination.

This study aimed to determine the mean score for knowledge and the smoking refusal skill and to compare them between the control and intervention groups. The sample size calculation was done for each objective and the one that yielded the biggest sample size was taken as the study sample size. PS software (independent t test) for comparing 2 mean was utilized to calculate sample size for the mean score for respective objectives. Smoking refusal skill yielded the largest sample size, with standard deviation of refusal skill score of 12.46 (29) and detectable difference of 6. Considering the non-response rate of 20%, the calculated sample size of 83 subjects per group was taken as the study sample size.

The lists of students from the two selected schools were obtained from the respective schools’ administration. Students were eligible to participate if they were form one or form two students (which corresponds to age 13 and 14 years old), agreed to participate in the study and consented by the parents or legal guardians and assent to the study. Exclusion criteria were special class students and self-reported smokers. Students who fulfilled the inclusion and exclusion criteria were randomly selected using simple random sampling which was computer-generated based on the number of the eligible respondents. Parental consents and student informed assents were obtained from each respondent and their parents or guardian prior to the study. All procedures in the study were approved by research and ethics committee of School of Medical Science, Health Campus, Universiti Sains Malaysia (USM/JEPeM/16010021).

The questionnaires were adapted and modified with consent from the questionnaires used in a study in Taiwan (Lee et al., 2007) and the Global Youth Tobacco Survey (GYTS) core questionnaire (Centers for Disease Control Prevention, 2012). The modification was done based on literature review and expert opinions with the aim of determining the relevant items for content validity. The questionnaires were divided into three sections: socio-demographic data, knowledge on smoking and smoking refusal skill.

*a. Socio-demographic data*

This section consists of seven questions including gender, age, current living arrangement, parents’ educational level and environmental smoking.

*b. Knowledge on smoking*

This section consists of 23 questions regarding knowledge on smoking prevalence, smoking harmful effects and law or rules related to smoking with answers of ‘yes’, ‘no’ and ‘don’t know’. Each correct answer worth one point, no point was given or deducted for incorrect answer or ‘don’t know’. The total score for this section was 23.

*c. Smoking refusal skill*

This section consists of 8 techniques of refusing cigarettes. A 5-point Likert scale was used ranging from scores of ‘*1 - very impossible*’, ‘*2 - impossible*’, ‘*3 - unsure*’, ‘*4 - possible*’ and ‘*5 - very possible*’. The total score in this section was 40. The higher the score, the higher the probability of the refusal technique being used. 

The knowledge and refusal skill score were then converted into mean score and mean standard deviation (SD). Then, the results were compared between the control group and the intervention group. 

*Smoking prevention module*

This module consisted of three main activities which took around three hours to partake. The module was adapted, modified and revised by a group of Universiti Sains Malaysia’s lecturers to suit our aim to provide knowledge on smoking harmful effects and smoking refusal skills. It was prepared in Malay and consists of three main activities which include ‘*smoker’s body*’ activity, a video session and ‘*I don’t want to smoke*’ activity. During the ‘*smoker’s body*’ activity, the participants were encouraged to discuss among the group members about the harmful effects of smoking and to illustrate them into simple images. Then the groups’ representative presented their work followed by comments from the facilitator cum co-investigator. Following this activity, a 5-minute video which educate on smoking harmful effects and law or rules related to smoking in Malaysia was shown to the participants. The third activity, ‘*I don’t want to smoke*’ was a role play activity. In this session, the participants were encouraged to actively discuss on how to resist smoking invitation guided by a scenario card and facilitator. After 10 minutes of discussion, every group were given 15 minutes each to act out the given scenario. They were encouraged by the facilitator to use straight body posture, use clear and precise words, be confident while talking and make good eye contacts. Finally, the participants were shown another 3-minute video on how to refuse smoking invitation. The video content was a role play by a group of adolescents that explained eight examples of refusing smoking invitation which were advising the dangers of smoking, using health problem as an excuse, changing the topic of conversation, using own reasons, advising the smoking disadvantages, proposing advantageous activity, using arguments to oppose and firmly reject the invitation. The contents and techniques used in this video are based on the effective verbal and non-verbal skills that are recommended by literatures.

*Protocol*

Permissions were obtained from Ministry of Education, Ministry of Health and Kelantan State Education Department before the study was carried out. Two secondary schools from different suburbs were selected. One was appointed as a control group while the other was designated as intervention group. The schools were visited several times before the completion of the study.

*First visit*

The school principals and counsellors were briefed regarding the study. The timing of the programme was also discussed and agreed upon to avoid interruption to their class schedule. The lists of all form one and form two students from both schools were also obtained from the school administration. Declaration forms with specific coding were then prepared according to the lists.

*Second visit*

Screening of participants was carried out. All form one and form two students were gathered at the school hall. The students were then informed that they needed to state whether they were smokers or otherwise in the declaration form. This is the salient aspect of the study as we needed to exclude smokers from participating. The students were also informed that even if they were not smokers, it did not necessarily mean that they would be automatically selected to participate in the study. This is because subjects would be randomly chosen by a computer-generated system. For ethical and confidential purposes and to ensure maximum participation, the students were assured that the declaration forms would never be revealed to their teachers, parents and friends. The declaration forms were secured in a sealed box that could only be opened by the researcher. The smokers were identified by the coding created based on the name lists.

After ruling out smokers and those who were not present at school, the complete list of non-smokers was recorded. A total of 83 students from each school were then recruited from this list using computer-generated simple random sampling. The students were divided into six groups, consist of 13 to 14 students each. The list of selected students was shared with the counsellors. The students were then briefed and provided with informed assent forms and parental consents. 

For ethical purposes, self-proclaimed smokers were approached personally. They were counselled and convinced to inform their parents to consider further assistance to quit smoking. Unfortunately, none of them agreed to inform their parents.

*Third visit*

During this visit, both groups of students were gathered in the school hall and requested to answer the pre-test questionnaire based on their general knowledge which they might have received from social media or during learning process at school. Students were placed in exam like position so they would not be able to discuss with their peers.

The intervention group went through the smoking prevention module after the completion of pre–test questionnaire.

*Fourth visit (two weeks after the third visit)*

During this visit, the study participants were again gathered to answer the same questionnaire (post-test).

*Fifth visit (eight weeks after the third visit)*

Once more, participants were required to answer the questionnaire. Students from the control group were given the smoking intervention module upon completion of the questionnaire.

*Statistical analysis*

Data were entered and analyzed using “Statistical Package for Social Sciences (SPSS)” version 22 (SPSS Inc., Chicago, IL, USA). Data were checked and cleaned before analysis. The comparison of mean score of knowledge between control and intervention group and comparison of smoking refusal skills between control and intervention group at baseline, two weeks and two weeks post intervention were analyzed using repeated measure ANCOVA. 

## Results

*Characteristics of the respondents*

A total number of 166 non-smoker adolescents age 13 -14 years old participated in the study. Both intervention and control groups had equal number of participants (83) and the gender distribution were almost equal (Table 1). The majority of them have parents who had formal educations up to secondary level. Most of the students had observed someone smoking at home and claimed to see people smoking most of the days in their homes. The number for those exposed to smoking at home is almost similar between the two groups. The majority of them do not have close friends who smoke. Those in the intervention groups are more exposed to active smoker (53%) compared to those in the control groups (47%). Detail socio-demographic characteristics of the respondents are presented in Table 1.

*Knowledge on smoking*

The mean (SD) baseline knowledge score for control and intervention group were 12.23(3.893) and 12.45(3.697) respectively (p = 0.713) (Table 2). The comparison of score of knowledge on smoking and its harmful effects between control and intervention group is presented in Table 3. There is a significant difference in crude mean and estimated marginal means (p <0.001) of knowledge score between intervention and control group after controlling for gender. 

*Smoking refusal skill*

The mean (SD) baseline refusal skills score for control and intervention group were 30.89 (6.164) and 28.0 2 (6.241) respectively (p=0.003) (Table 4). 

The comparison of score of smoking refusal skills between control and intervention group is presented in Table 5. There is a significant difference in crude mean and estimated marginal means (p = 0.005) of smoking refusal skills score between intervention and control group after controlling for gender.

**Table 1:** Socio-Demographic Characteristics of the Subjects

Variables	Control group mean (SD) ͣ	(n = 83) n ( % )	Intervention group mean (SD) ͣ	(n = 83) n (%)	p value
Age	13.4 (0.490)		13.5 (0.501)		0.087^b^
Gender					
Male		41 (49.4)		43 (51.8)	0.756^c^
Female		42 (50.6)		40 (48.2)	
Father’s education level					
Primary & secondary		40 (48.2)		36 (43.4)	0.823^c^
Tertiary		12 (14.5)		13 (15.7)	
Don’t know		31 (37.3)		34 (41.0)	
Mother’s education level					
Primary & secondary		41 (50.0)		29 (34.9)	0.147^c^
Tertiary		16 (19.5)		21 (25.8)	
Don’t know		25 (13.5)		33 (39.8)	
Observe people smoking at home					
Most of the day		37 (44.6)		39 (47.0)	0.795^c^
Sometimes		13 (15.7)		10 (12.0)	
Nobody smokes at home		33 (39.8)		34 (41.0)	
Number of close friends that smoke					
None		44 (53.0)		39 (47.0)	0.710^c^
Some		32 (38.6)		35 (42.2)	
Most of them		7 (8.4)		9 (10.8)	

^a^, Standard deviation; ^b^, t test;^ c^, Chi square

**Table 2:** Baseline Score on Knowledge on Smoking and Its Harmful Effects

Variables	Control group (n=83)	Intervention group (n=83)	p value
	mean (SD)	mean (SD)	
Baseline knowledge score	12.23 (3.893)	12.45 (3.697)	0.713

Independent t test

**Table 3:** Comparison in Mean Score Difference of Knowledge on Smoking and Its Harmful Effects among Intervention and Control Group Based on Time (Time-treatment Interactions)

Group	N	Desc Mean^a^ (SD)^b^			EMM^c^ (95% CI)^d^			p value
		Week 0	Week 2	Week 8	Week 0	Week 2	Week 8	
Intervention	83	12.5	15.4 (4.73)	17.4	12.47	15.45	17.38	<0.001
		(3.70)		(4.71)	(11.67, 13.27)	(14.56, 16.33)	(16.44, 18.31)	
Control	83	12.2	12.7 (3.93)	13.1	12.21	12.63	13.16	
		(3.89)		(3.90)	(11.41, 13.01)	(11.74, 13.52)	(12.22, 14.09)	

Repeated measures ANCOVA; ^a^, Descriptive mean; ^b^, Standard deviation; ^c^, Estimated Marginal Means; ^d^, Confidence Interval

**Table 4:** Baseline Smoking Refusal Skill Score Comparison between Control and Intervention Group

Variables	Control group (n=83) mean (SD)	Intervention group (n=83) mean (SD)	p value
Baseline refusal skill score	30.89 (6.164)	28.02 (6.241)	0.003

Independent t test

**Table 5:** Comparison in Mean Score Difference of Smoking Refusal Skill among Intervention and Control Group Based on Time (Time-treatment Interactions)

Group	N	Desc Meana (SD)^b^			EMMc (95% CI)^d^			p value
		Week 0	Week 2	Week 8	Week 0	Week 2	Week 8	
Intervention	83	28.02 (6.24)	29.16 (6.39)	30.70 (6.00)	28.07 (26.80, 29.34)	29.22 (27.89, 30.54)	30.72 (29.41, 32.03)	0.005
Control	83	30.89 (6.16)	30.06 (6.76)	30.77 (6.14)	30.84 (29.57, 32.11)	30.00 (28.67, 31.33)	30.75 (29.44, 32.06)	

Repeated measures ANCOVA; ^a^, Descriptive mean;^ b^, Standard deviation; ^c^, Estimated Marginal Means; ^d^, Confidence Interval

## Discussion

Malaysia is aiming for the “*End Game*” by 2045. To achieve this, every level of the community has to be tackled and preventive method need to be strengthened. Among the important stakeholder are the adolescents. In order to ensure that more adolescents are not ensnared in this addictive behavior, preventive modules need to be developed and executed. One important aspect in prevention is to strengthen the basic knowledge on the harmful effects of smoking itself and to instill a skill on how to refuse when faced with the invitation to smoke. The findings from current study supported the positive effects of smoking prevention module on the level of knowledge on smoking and its harmful effects and smoking refusal skills among the adolescents. 

*Knowledge on smoking and its harmful effects*

The harmful effects of smoking are widely discussed and accessible to all level of people in the community. Ministry of Health as a representative of the government all over the world and nongovernmental organizations had been very passionate in spreading the information on the bad effects of smoking. Thus, it is expected that the adolescents have some knowledge on the harmful effects of smoking. (Fathelrahman et al., 2013, Myint and Yee, 2016, Norbanee et al., 2006, TECMA 2016). However, the assessment on the success of the campaign is sometimes neglected. One of the objectives of this study was to assess this as reflected by the baseline knowledge on smoking and its harmful effects. In this study, the mean score of knowledge was about half of what is expected. Both groups have similar knowledge score of about 12 out of the total score of 23. The fact that both groups were of similar age and have similar baseline knowledge is important to ensure that the intervention really able to increase the knowledge among the adolescents and the outcome is comparable.

The effectiveness of the module in increasing the knowledge can be seen at two weeks and eight weeks post intervention assessment and increasing as the time went by. Although similar increment was seen in the control group, the changes was minimal. This could be explained by the possibility that after being exposed to the questionnaire, the adolescents’ interest and attention on the effects of smoking increased, thus, inducing them to be more aware of the effects, thus, increasing their current knowledge. This was the theory behind the increment in the knowledge of the control group who did not received additional information, other than what they could gather from their surroundings. The effectiveness of smoking prevention module to increase knowledge on smoking deleterious effect had been shown in many studies around the world (Lee et al., 2007, Carreras et al., 2016, Maruska et al., 2016, St Germain et al., 2017). However, since reception of adolescents may vary, it is very important to deliver it in the way which is acceptable to the local community and the target age group. Studies found that the use of video had been shown to improve the efficacy of interventions or educations (Harskamp et al., 2007; Walma Van Der Molen and Voort, 2000; Wilson et al., 2010). Another study found that a video session improved the knowledge of the participants especially the immediate recalls. Other methods such as printed materials enhanced the efficacy of the video session (Wilson et al., 2010). Similarly, our module used video to intensify the delivery of the information to the adolescents and to ensure the receptive and sustainability of the knowledge. In the age where technology and gadgets are the “*in*” thing, the delivery of the information has to be updated. The classroom type lectures and pamphlets will not interest the audience especially the adolescents anymore. 

*Smoking refusal skills*

Most adolescents were ensnared in tobacco smoking habit because they were influenced by their peers or adults around them. Thus, equipping them with refusal skills is an important step in preventing them from being dragged into the habit. One study had proved that refusal skill was one of the main determinants of smoking initiation and better refusal skills lowered the likelihood of being involved in this deviant behavior (Karimy et al., 2013). In this study, the students were all non-smokers, thus, it is safe to assume that all of them had some refusal skills to be able to resist the temptation to smoke which they may have gathered from their surroundings. There has not been any formal teaching in education syllabus to combat smoking, thus, different schools and teachers may have used different styles and initiatives in delivering information related to smoking and how to resist this harmful activity which will result in different outcomes. The control group in this study started with significantly better refusal skills compared to the intervention group. 

However, post intervention, the smoking refusal skills score among the intervention group’s respondents were significantly higher as compared to the control group in the second and eighth week of the study. This indicated that the smoking prevention module significantly improved the smoking refusal skill among the respondents. These findings were parallel with previous studies which showed that skills intervention significantly increased the smoking refusal skills among the adolescents (Brown et al., 2007, Lee et al., 2007, Maruska et al., 2016). 

Hence, this study had highlighted that, besides fortifying knowledge related to smoking among adolescents, it is equally important to strengthen their smoking refusal skills in order to prevent them from falling prey to the notorious habit. Since adolescence was well known to be a very sensitive transition period, where the actions may have long-lasting effect, it is very important that the refusal skills are instilled within them as early as possible. 

In conclusion, this quasi intervention study showed that smoking prevention module increased the level of knowledge on smoking and its harmful effects and smoking refusal skills among the secondary school students. The module used has to be tailored to the target group and has to be modified according to age and local practice.

*Limitation and Recommendation*

The study was limited to one district and involved a small number of students. We recommend larger scale study be carried out to in different regions of the country, so the strength of the study may be demonstrated at bigger scale. We also advocated that the module is used as one of the standard modules to improve the current method of teaching in delivering knowledge related to harmful effects and laws related to smoking and that smoking refusal skill to be taught to all secondary school students. It is hoped that all this may help us to eventually see the realization of the “*Malaysia-Tobacco End Game*” in 2045.

*Ethical Consideration*

Ethical clearance was obtained from Human Research Ethics Committee, Universiti Sains Malaysia (USM/JEPeM/16010021). The data for this study was presented as collective and not by individual information to preserve the confidentiality of its participants. The self-proclaimed smokers were approached personally. They were counselled and convinced to inform their parents to consider further assistance to quit smoking. Unfortunately, none of them agreed to inform their parents.
